# Biopsy of cervical lymph node does not impact the survival of nasopharyngeal carcinoma

**DOI:** 10.1002/cam4.4204

**Published:** 2021-08-12

**Authors:** Shi‐Ping Yang, Ji‐Fang Li, Ping Zhou, Chen‐Lu Lian, Dan‐Xia Chen, Zhao‐Jun Li, San‐Gang Wu

**Affiliations:** ^1^ Department of Radiation Oncology Hainan General Hospital (Hainan Affiliated Hospital of Hainan Medical University) Haikou People's Republic of China; ^2^ Department of Clinical Nutrition Hainan General Hospital (Hainan Affiliated Hospital of Hainan Medical University) Haikou People's Republic of China; ^3^ Department of Radiation Oncology The First Affiliated Hospital of Xiamen University Xiamen People's Republic of China

**Keywords:** clinical practice, histology, lymph node biopsy, nasopharyngeal carcinoma, prognosis

## Abstract

**Purpose:**

The optimal practice regarding cervical lymph node biopsy (CLNB) remains to be defined to provide the best clinical management in nasopharyngeal carcinoma (NPC). This study aimed to investigate the effect of CLNB on the survival of NPC patients.

**Methods:**

Patients diagnosed with NPC from 2004 to 2015 were identified using the Surveillance, Epidemiology, and End Results database. Multivariate logistic regression, Kaplan–Meier method, Cox proportional hazards regression analysis, and propensity score matching (PSM) were used to determine the factors associated with CLNB and prognostic effect of CLNB of NPC.

**Results:**

We included 1903 patients in this study. There were 321 (16.9%) and 1582 (83.1%) patients with and without CLNB, respectively. The percentage of CLNB was 19.4% in 2004 and was decreased to 8.6% in 2015 (*p* = 0.044). Patients diagnosed in later years (*p* = 0.008), older age (*p* < 0.001), Chinese (*p* = 0.002), advanced tumor stage (*p* < 0.001), and early nodal stage (*p* = 0.003) were less likely to receive additional CLNB. In patients who received additional CLNB, the 5‐years NPC‐specific survival (NPCSS) was 83.6%, which was similar to patients without CLNB (80.1%, *p* = 0.159). In addition, a similar 5‐years NPCSS was found between those receiving biopsy or aspiration of regional lymph node and those receiving lymph node resection (*p* = 0.584). There were 187 pairs of patients who were completely matched using PSM, the multivariate prognostic analyses indicated that the receipt of CLNB was not associated with an inferior outcome in the PSM cohort (*p* = 0.349). Similar results were found after stratification by the year of diagnosis, race/ethnicity, and histology.

**Conclusion:**

Additional CLNB is not associated with an inferior survival outcome in NPC. Our study provides a reference for the clinical practice of NPC.

## Graphical abstract

The optimal practice regarding the cervical lymph node biopsy (CLNB) remains to be defined to provide the best clinical management in nasopharyngeal carcinoma (NPC). This study aimed to investigate the effect of CLNB on the survival of NPC patients using a population‐based cohort. Our study suggests that additional CLNB is not associated with increased risk of cancer‐specific mortality of NPC.

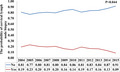

## 1 INTRODUCTION

Nasopharyngeal carcinoma (NPC) is frequently developed in Southern China, Northern Africa, and Alaska populations. The adjusted incidence in the Southern China population is between 20 and 50 per 100,000 inhabitants annually but is only 0.5–1 per 100,000 annually in Caucasian populations.^1^, ^2^, ^3^ Approximately 85% of NPC patients have cervical lymph node metastasis (CLNM) at the initial diagnosis.^4^ Biopsy of the suspicious lesion is critical for the diagnosis of NPC. However, 3%–5% of patients have CLNM without apparent primary tumor lesions.^5^, ^6^ Cervical lymph node biopsy (CLNB) is a potential option for the definitive diagnosis of these patients. However, concerns were raised regarding the safety of CLNB due to the potential to cause tumor dissemination.^7^, ^8^ Prior study has shown that repeat nasopharyngeal biopsy before definitive radiotherapy may increase the risk of distant recurrence of NPC patients.^9^ Therefore, whether the CLNB in NPC patients will increase the risk of distant recurrence and mortality requires further research.

Several studies have attempted to answer this question in NPC patients,^10^, ^11^, ^12^, ^13^ but reached conflict conclusions. The introduction of intensity‐modulated radiotherapy (IMRT) into clinical management has made great improvements in the survival of NPC patients.^14^, ^15^, ^16^ Moreover, additional chemotherapy also decreased the incidence of distant metastasis and mortality of NPC.^17^ However, the current recommendation regarding the CLNB remains controversial in various clinical practice guidelines of NPC, including National Comprehensive Cancer Network (NCCN) Guidelines in 2020,^18^ European Society for Medical Oncology (ESMO)‐European Rare CANcer (EURACAN) Clinical Practice Guideline in 2021,^19^ Guidelines of Chinese Society of Clinical Oncology (CSCO) in 2020,^20^ Spanish Society of Medical Oncology (SEOM) Clinical Guideline in 2017,^21^ and the United Kingdom National Multidisciplinary Guidelines in 2016.^22^ Therefore, the optimal practice regarding CLNB in NPC remains to be defined to provide a higher level of evidence for clinical management. In light of this, the present study aimed to investigate the effect of CLNB on the prognosis of NPC patients using a population‐based cohort.

## 2 MATERIALS AND METHODS

### 2.1 Data source and patients

The Surveillance, Epidemiology, and End Results (SEER) program of the National Cancer Institute collects and publishes data regarding cancer incidence, the first course of treatment, and outcome in the United States (US), which covers approximately 35% of the U.S. population. The database used in our study was SEER Research Plus Data.^23^ We included patients with the following inclusion criteria: (1) diagnosed with NPC between 2004 and 2015; (2) World Health Organization (WHO) I‐III subtypes (WHO I, keratinizing squamous cell carcinoma; WHO II, non‐keratinizing squamous cell carcinoma; WHO III, undifferentiated non‐keratinizing squamous cell carcinoma); (3) node‐positive disease with or without CLNB before radiotherapy; (4) received beam irradiation with or without chemotherapy. We excluded patients with metastatic (M) stage, unavailable for tumor (T) and nodal (T) stage, and received CLNB after radiotherapy. The Institutional Review Board approval of the present study was not required due to the de‐identified patient information in the SEER dataset.

### 2.2 Variables

The following demographic and clinicopathological factors were extracted from the SEER dataset: year of diagnosis, race/ethnicity, gender, age, histology, T stage, N stage, chemotherapy use, and the use of CLNB. The sixth American Joint Committee on Cancer (AJCC) TNM staging system was used in this study.^24^ The primary end‐point of this study was NPC‐specific survival (NPCSS), which was computed as death due to NPC during the follow‐up.

### 2.3 Statistical analysis

The proportions of categorical data were compared using the Chi‐square test. The independent predictive factors associated with the utilization of CLNB were determined using multivariate logistic regression. The NPCSS curves were analyzed by the Kaplan–Meier method and compared using the log‐rank test between groups. A 1:1 propensity score matching (PSM) method was used to balance the patient characteristics using the following variables, the year of diagnosis, age, gender, race/ethnicity, histology, T stage, N stage, and chemotherapy use. Cox proportional hazards regression analysis was performed to investigate the independent prognostic factors associated with NPCSS. Sensitivity analyses focused on the year of diagnosis, histology, and race/ethnicity were performed. All statistical analyses were performed using SPSS version 22.0 (SPSS Inc.). A *p*‐value less than 0.05 was considered statistically significant.

## 3 RESULTS

### 3.1 Patients' characteristics

We included 1903 patients in this study (Table 1). Of these patients, 62.0% (*n* = 1179) of patients were aged ≥50 years, 69.9% (*n* = 1330) were male. Regarding race/ethnicity, 47.5% (*n* = 904), 12.6% (*n* = 209), 15.3% (*n* = 291), and 24.6% (*n* = 469) were White, Black, Chinese, and other race, respectively. In addition, there were 765 (40.2%), 616 (32.4%), and 522 (27.4%) patients were WHO I, WHO II, and WHO III subtypes, respectively. Regarding the sixth TNM staging, 26.0% (*n* = 494), 39.1% (*n* = 744), 21.0% (*n* = 399), and 14.0% (*n* = 266) of them had stage IIB, III, IVA, and IVB diseases, respectively.

**TABLE 1:** The patient baseline characteristics before and after propensity score matching

Variables	Before PSM				After PSM			
	*N*	No CLNB (%)	CLNB (%)	*p*	*N*	No CLNB	CLNB	*p*
Year of diagnosis								
2004–2007	561	446 (28.2)	115 (35.8)	0.003	142	71	71	1
2008–2011	642	530 (33.5)	112 (34.9)		136	68	68	
2012–2015	700	606 (38.3)	94 (29.3)		94	48	48	
Age (years)								
<50	724	575 (36.3)	149 (46.4)	0.001	142	71	71	1
≥50	1179	1007 (63.7)	172 (53.6)		232	116	116	
Gender								
Male	1330	1105 (69.8)	225 (70.1)	0.930	290	145	145	1
Female	573	477 (30.2)	96 (29.9)		84	42	42	
Race/ethnicity								
White	904	735 (46.5)	169 (52.6)	0.038	234	117	117	1
Black	239	205 (13.0)	34 (10.6)		30	15	15	
Chinese	291	256 (16.2)	35 (10.9)		28	14	14	
Other	469	386 (24.4)	83 (25.9)		82	41	41	
Histology								
WHO I	765	629 (39.8)	136 (42.4)	0.239	172	86	86	1
WHO II	616	525 (33.2)	91 (28.3)		94	47	47	
WHO III	522	428 (27.1)	94 (29.3)		108	54	54	
T stage								
T1	521	370 (23.4)	151 (47.0)	<0.001	160	80	80	1
T2	525	431 (27.2)	94 (29.3)		116	58	58	
T3	413	374 (23.6)	399 (12.1)		46	23	23	
T4	444	407 (25.7)	37 (11.5)		52	26	26	
N stage								
N1	876	742 (46.9)	134 (41.7)	0.013	166	83	83	1
N2	762	636 (40.2)	126 (39.3)		158	79	79	
N3	265	204 (12.9)	61 (19.0)		50	25	25	
Chemotherapy								
No	100	83 (5.2)	17 (5.3)	0.971	8	4	4	1
Yes	1803	1499 (94.8)	304 (94.7)		366	183	183	

Abbreviations: CLNB, cervical lymph node biopsy; N, nodal; PSM, propensity score matching; T, tumor; WHO, World Health Organization.

**FIGURE 1:**
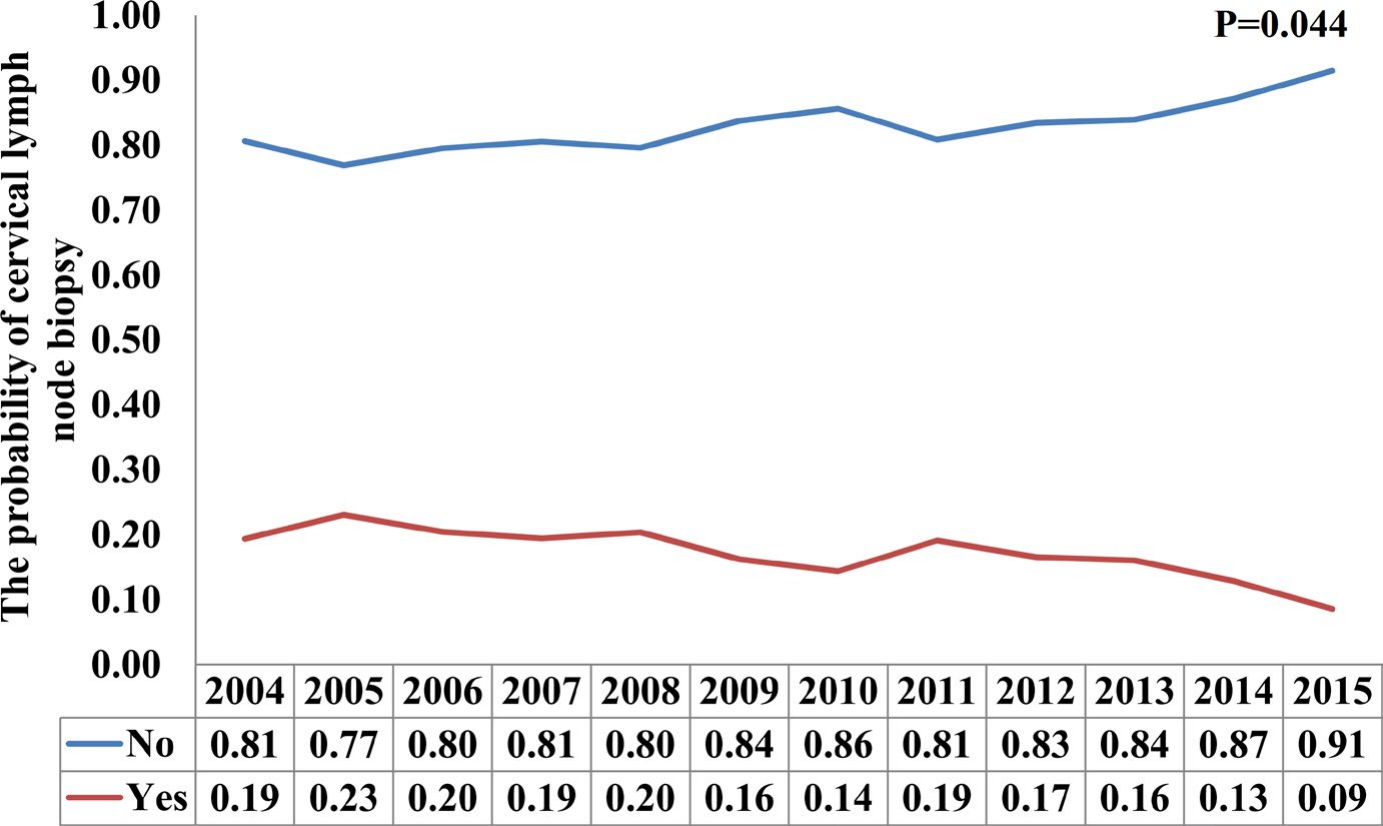
The percentage of the utilization of cervical lymph node biopsy from 2004 to 2015

Of these patients, there were 321 (16.9%) patients had CLNB, and 1582 (83.1%) patients had no CLNB. In the 321 patients who had CLNB, 104 (32.4%) receiving biopsy or aspiration of regional lymph node and 217 (67.6%) patients undergoing lymph node resection. Patients diagnosed in early years (*p* = 0.003), younger age (*p* = 0.001), White race (*p* = 0.038), early T stage (*p* < 0.001), and advanced N stage (*p*=0.013) were more likely to receive CLNB. The percentage of cervical lymph node resection or biopsy was 19.4% in 2004 and was decreased to 8.6% in 2015 (*p* = 0.044) (Figure 1). Moreover, there were 94.7% (*n* = 1803) of patients received chemotherapy, and patients with additional CLNB were not associated with a higher percentage of chemotherapy use (*p* = 0.971).

### 3.2 Predictors of additional CLNB

Multivariate logistic regression was used to determine the predictive factors associated with the use of CLNB (Table 2). The results showed that patients diagnosed in later years (diagnosed in 2012–2015 vs. diagnosed in 2004–2007, odds ratio [OR] 0.656, 95% confidence interval [CI] 0.480–0.895, *p* = 0.008), older age (aged ≥50 years vs. aged <50 years, OR 0.624, 95% CI 0.483–0.806, *p* < 0.001), Chinese (Chinese vs. White, OR 0.521, 95%CI 0.348–0.781, *p* = 0.002), and advanced T stage (as the T stage increases, fewer patients undergo CLNB, all *p* < 0.001) were less likely to receive additional CLNB. Moreover, patients diagnosed with the N3 stage were more likely to receive CLNB (N3 vs. N1, OR 1.733, 95% CI 1.211–2.481, *p* = 0.003).

**TABLE 2:** Predictive factors associated with the use of CLNB in NPC

Variables	OR	95% CI	*p*
Year of diagnosis			
2004–2007	1		
2008–2011	0.941	0.695–1.273	0.693
2012–2015	0.656	0.480–0.895	0.008
Age (years)			
<50	1		
≥50	0.624	0.483–0.806	<0.001
Gender			
Male	1		
Female	0.946	0.720–1.245	0.694
Race/ethnicity			
White	1		
Black	0.753	0.496–1.144	0.184
Chinese	0.521	0.348–0.781	0.002
Other	0.887	0.652–1.205	0.442
Histology			
WHO I	1		
WHO II	0.793	0.582–1.080	0.140
WHO III	0.996	0.726–1.367	0.981
T stage			
T1	1		
T2	0.505	0.375–0.681	<0.001
T3	0.243	0.165–0.358	<0.001
T4	0.216	0.146–0.320	<0.001
N stage			
N1	1		
N2	1.169	0.885–1.543	0.271
N3	1.733	1.211–2.481	0.003

Abbreviations: CI, confidence interval; CLNB, cervical lymph node biopsy; N, nodal; NPC, nasopharyngeal carcinoma; OR, odds ratio;T, tumor; WHO, World Health Organization.

### 3.3 Survival and prognostic analyses

**FIGURE 2:**
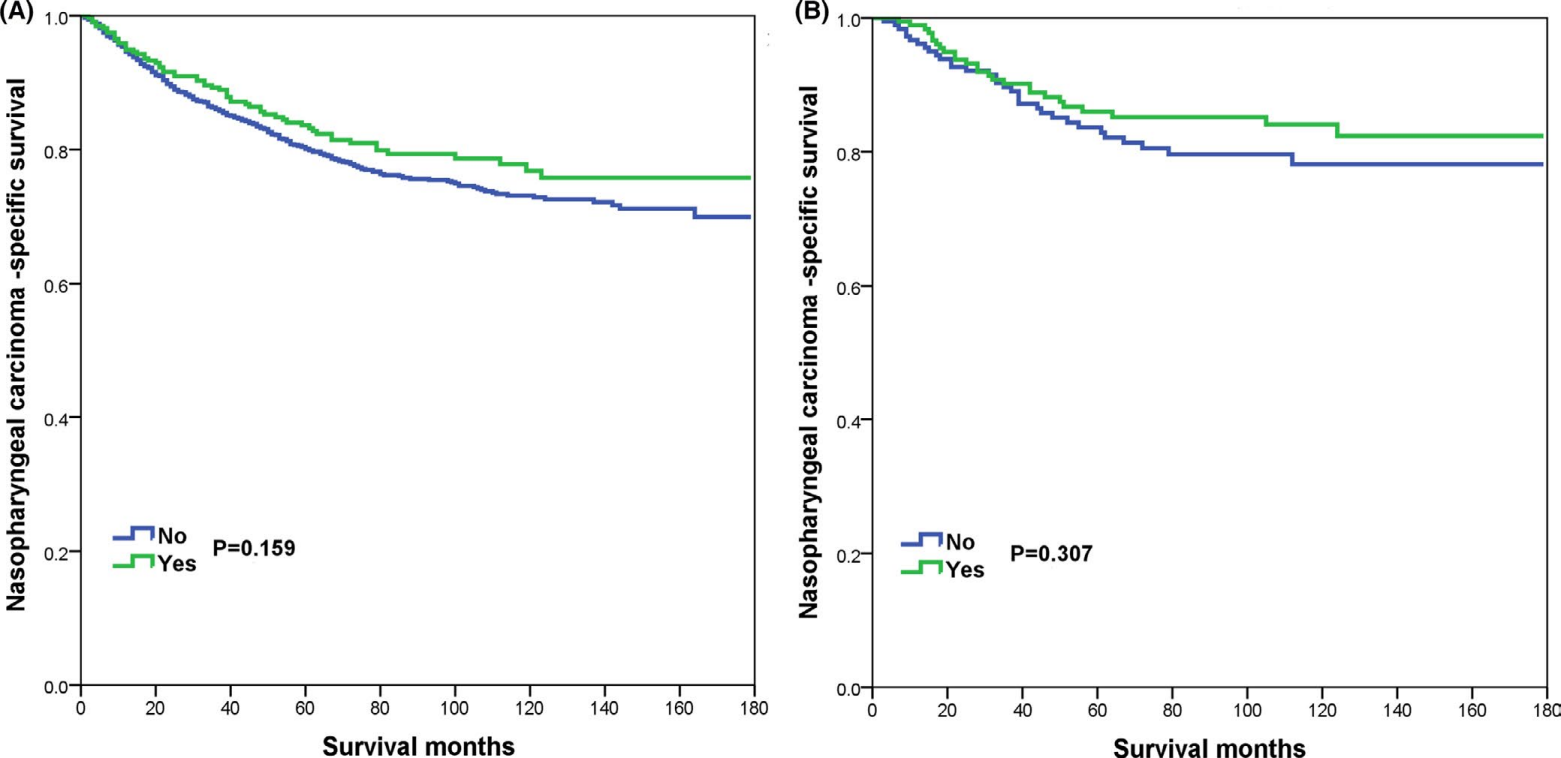
The effect of cervical lymph node biopsy on nasopharyngeal carcinoma‐specific survival before and after propensity score matching

With a median follow‐up time of 64 months (range, 0–179 months), 391 patients died with NPC. The 5‐years and 10‐years NPCSS were 80.7% and 73.8%, respectively. In patients who received additional CLNB, the 5‐years NPCSS was 83.6%, which was similar to patients without CLNB (80.1%, *p* = 0.159) (Figure 2A). Regarding those treated with CLNB, the 5‐years NPCSS was 83.4% in those receiving biopsy or aspiration of regional lymph node and 82.0% in those receiving lymph node resection (*p* = 0.584). There were 187 pairs of patients who were completely matched using PSM (Table 1) and the results also showed comparable NPCSS between those with or without additional CLNB (83.7% vs. 86.0%, *p* = 0.307) (Figure 2B).

In the PSM cohort, the multivariate Cox proportional hazards regression analysis was used to determine the independent prognostic factors related to NPCSS (Table 3). The results showed that the N stage was the independent prognostic factor related to NPCSS. However, similar survival was found between those treated with or without additional CLNB (hazard ratio [HR] 1.279, 95% CI 0.764–2.143, *p* = 0.349).

**TABLE 3:** Multivariate prognostic analysis in the study cohort after propensity score matching

Variables	HR	95% CI	*p*
Year of diagnosis			
2004–2007	1		
2008–2011	1.199	0.615–2.338	0.594
2012–2015	1.429	0.672–3.036	0.354
Age (years)			
<50	1		
≥50	1.675	0.966–2.906	0.066
Gender			
Male	1		
Female	1.095	0.586–2.047	0.775
Race/ethnicity			
White	1		
Black	0.972	0.376–2.515	0.953
Chinese	0.981	0.317–3.033	0.973
Other	0.815	0.412–1.611	0.556
Histology			
WHO I	1		
WHO II	0.892	0.467–1.701	0.728
WHO III	0.696	0.331–1.462	0.339
T stage			
T1	1		
T2	1.096	0.570–2.108	0.783
T3	1.277	0.537–3.036	0.581
T4	2.01	0.943–4.281	0.070
N stage			
N1	1		
N2	1.859	1.020–3.388	0.043
N3	3.434	1.703–6.925	0.001
CLNB			
No	1		
Yes	1.279	0.764–2.143	0.349
Chemotherapy			
No	1		
Yes	0.612	0.079–4.765	0.639

Abbreviations: CI, confidence interval; CLNB, cervical lymph node biopsy; HR, hazard ratio; N, nodal; T, tumor; WHO, World Health Organization.

### 3.4 Association of additional CLNB with survival after stratification by the year of diagnosis

**FIGURE 3:**
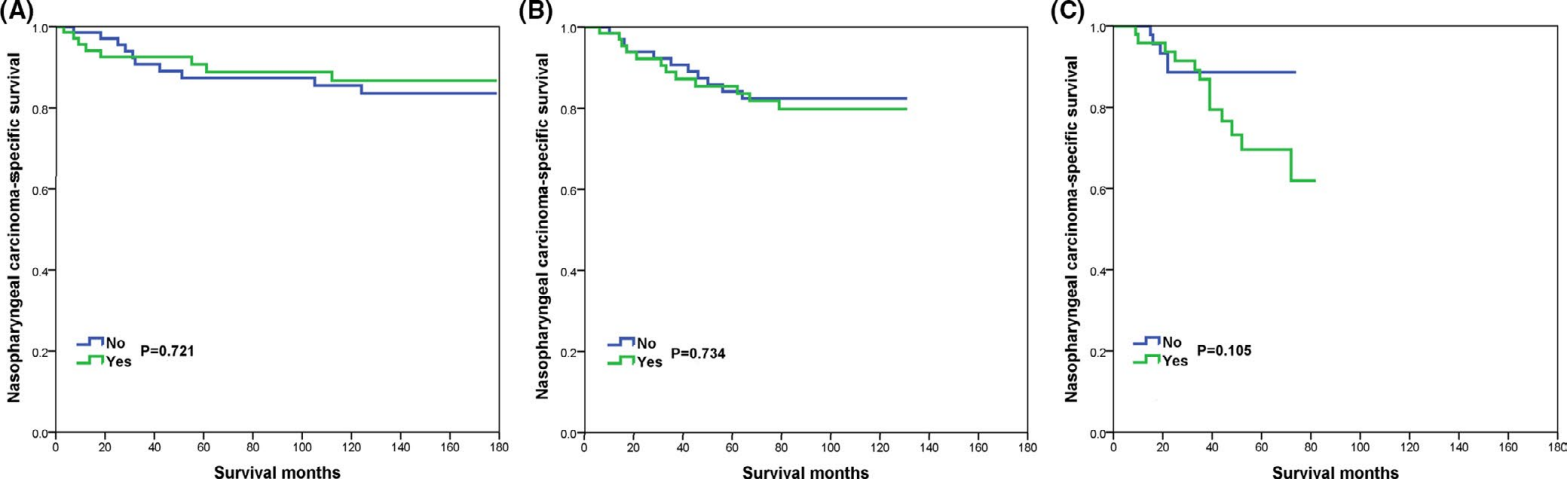
The effect of cervical lymph node biopsy on nasopharyngeal carcinoma‐specific survival in the study cohort after propensity score matching by stratification of the year of diagnosis (A, 2004–2007; B, 2008–2011; C, 2012–2015)

In the PSM cohort, the prognostic effect of additional CLNB on NPCSS by the year of diagnosis is summarized in Table 4. On multivariate Cox analysis, receipt of additional CLNB was not associated with an inferior survival outcome in those diagnosed in 2004–2007 (HR 0.859, 95% CI 0.333–2.219, *p* = 0.754), 2008–2011 (HR 1.129, 95% CI 0.491–2.598, *p* = 0.776), and 2012–2015 (HR 2.292, 95% CI 0.815–6.446, *p* = 0.116). The survival curves are listed in Figure 3A–C.

**TABLE 4:** Multivariate prognostic analysis in the study cohort after propensity score matching by stratification of the year of diagnosis, race/ethnicity, and histology

Variables	HR	95% CI	*p*
2004–2007^a^			
No CLNB	1		
CLNB	0.859	0.333–2.219	0.754
2008–2011^a^			
No CLNB	1		
CLNB	1.129	0.491–2.598	0.776
2012–2015^a^			
No CLNB	1		
CLNB	2.292	0.815–6.446	0.116
White^b^			
No CLNB	1		
CLNB	1.283	0.660–2.494	0.463
Black^b^			
No CLNB	1		
CLNB	0.404	0.056–2.943	0.371
Chinese^b^			
No CLNB	1		
CLNB	1.337	0.159–11.211	0.789
Other race^b^			
No CLNB	1		
CLNB	2.305	0.705–7.532	0.167
WHO I^c^			
No CLNB	1		
CLNB	1.336	0.635–2.814	0.445
WHO II^c^			
No CLNB	1		
CLNB	1.274	0.466–3.481	0.637
WHO III^c^			
No CLNB	1		
CLNB	1.184	0.390–3.590	0.766

Abbreviations: CI, confidence interval; CLNB, cervical lymph node biopsy;HR, hazard ratio; WHO, World Health Organization.

^a^ indicates adjustment of age, gender, race/ethnicity, histology, tumor stage, nodal stage, and chemotherapy.

^b^ indicates adjustment of the year of diagnosis, age, gender, histology, tumor stage, nodal stage, and chemotherapy.

^c^ indicates adjustment of the year of diagnosis, age, gender, race/ethnicity, tumor stage, nodal stage, and chemotherapy.

### 3.5 Association of additional CLNB with survival after stratification by race/ethnicity

**FIGURE 4:**
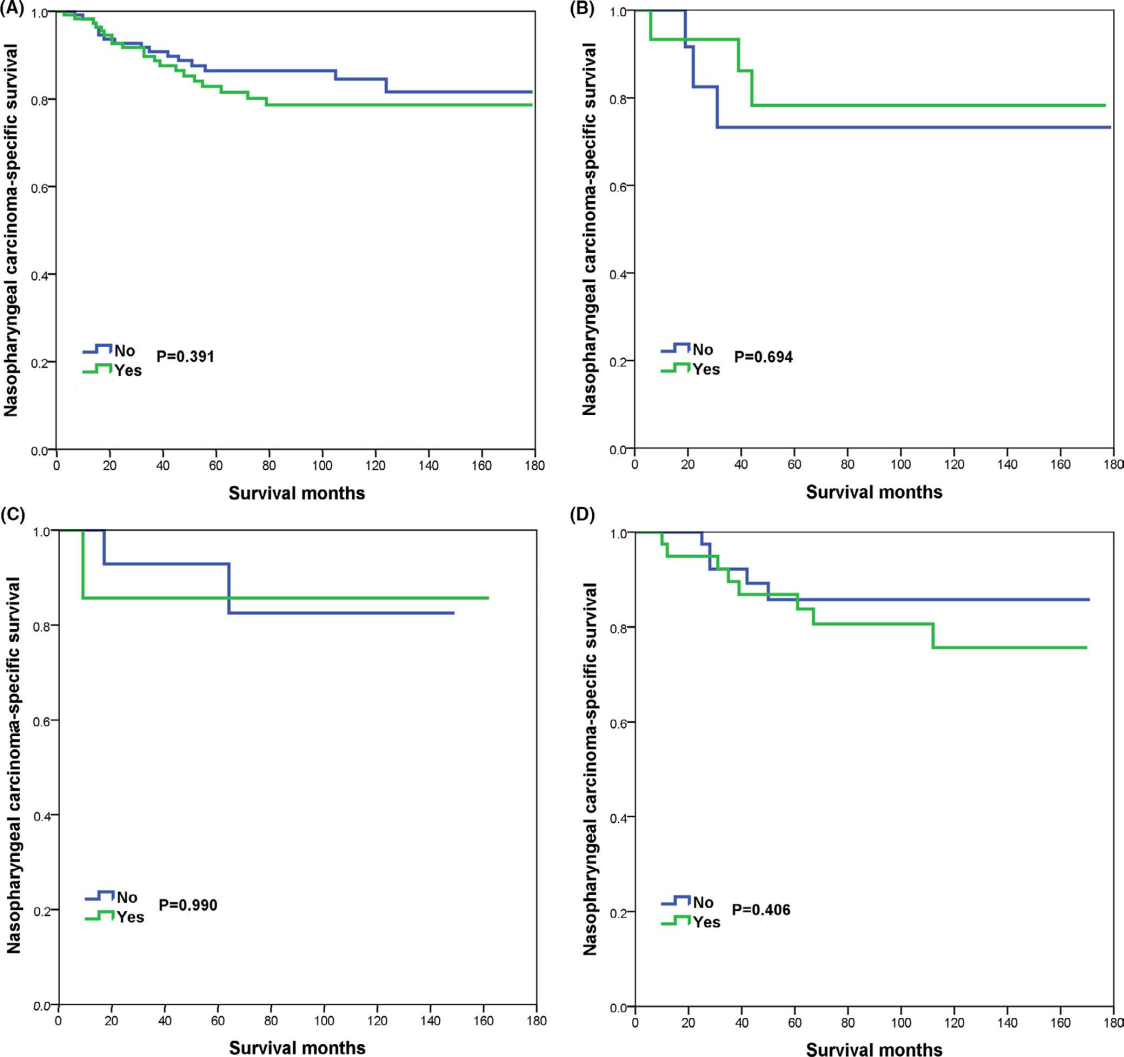
The effect of cervical lymph node biopsy on nasopharyngeal carcinoma‐specific survival in the study cohort after propensity score matching by stratification of race/ethnicity (A, White; B, Black; C, Chinese; D, Other race)

In the PSM cohort, the prognostic effect of additional CLNB on NPCSS by different races/ethnicities is summarized in Table 4. On multivariate Cox analysis, the receipt of additional CLNB was not associated with an inferior survival outcome in White (HR 1283, 95% CI 0.660–2.494, *p* = 0.463), Black (HR 0.404, 95% CI 0.056–2.943, *p* = 0.371), Chinese (HR 1.337, 95% CI 0.159–12.211, *p* = 0.789), or other race/ethnicity (HR 2.305, 95%CI 0.705–7.532, *p* = 0.167) patients. The survival curves are listed in Figure 4A–D.

### 3.6 Association of additional CLNB with survival after stratification by histology

**FIGURE 5:**
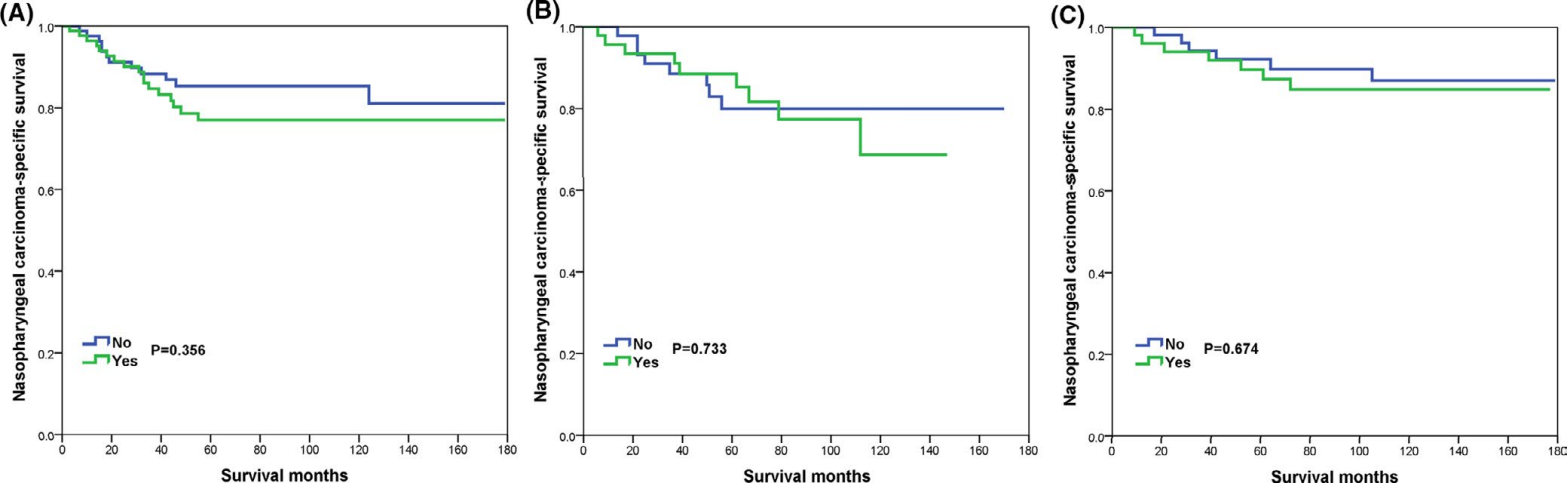
The effect of cervical lymph node biopsy on nasopharyngeal carcinoma‐specific survival in the study cohort after propensity score matching by stratification of histology (A, WHO I; B, WHO II; C, WHO III)

In the PSM cohort, the prognostic effect of additional CLNB on NPCSS by different histologies is summarized in Table 4. On multivariate Cox analysis, receipt of additional CLNB was not associated with an inferior survival outcome in WHO I (HR 1.336, 95% CI 0.635–2.814, *p* = 0.445), WHO II (HR 1.274, 95% CI 0.466–3.481, *p* = 0.637), or WHO III (HR 1.184, 95%CI 0.390–3.590, *p*=766) cancers. The survival curves are listed in Figure 5A–C.

## 4 DISCUSSION

In the present study, we used population‐based data from the SEER program to determine the prognostic effect of additional CLNB in NPC patients. Our results showed that the addition of CLNB did not associate with a higher risk of mortality of NPC patients. Similar findings were found after stratification by the year of diagnosis, race/ethnicity, and histology.

The progress of imaging technology, including positron emission tomography/computer tomography and magnetic resonance imaging (MRI), can more accurately measure the volume and extension of the tumor.^25^ In our study, we also found that the percentage of CLNB was 19.4% in patients diagnosed in 2004 and decreased to 8.6% for those diagnosed in 2015, which suggests the benefit of advances in imaging to discover potential cancer lesions. Patients with Epstein–Barr virus (EBV) positive in CLNM and unknown primary tumor are classified as T0 in the current AJCC NPC staging.^26^ In addition, some NPC patients were received CLNB at the time of diagnosis and then the nasopharyngeal biopsy was performed. Although the use of CLNB is critical in providing important additional diagnostic information in NPC, there have been concerns that CLNB will cause tumor cell dissemination.^27^, ^28^ In the current clinical practice, oncologists remain to find it hard to perform CLNB because there is still no consensus in various clinical practice guidelines.^18^, ^19^, ^20^, ^21^, ^22^ Fine needle aspiration biopsy of the cervical lymph node is recommended for diagnosing NPC or staging neck disease in the latest NCCN guidelines and United Kingdom National Multidisciplinary Guidelines, respectively.^18^, ^22^ The 2020 CSCO NPC guidelines recommend that for patients who cannot get a biopsy from the nasopharynx, CLNB is feasible.^20^ However, this procedure is not recommended in the latest version ESMO‐EURACAN Guidelines for NPC indicated that CLNB should be avoided because it may decrease the cure probability and have an adverse effect on late treatment sequelae. However, node dissection without capsular effraction or ultrasonography‐guided, transcutaneous tru‐cut biopsy is the best option if the primary tumor is not visible.^19^ In addition, the 2017 SEOM guideline also recommends that incisional CLNB will have a negative impact on the subsequent treatment.^21^ Our findings, therefore, go against some of the above clinical trial guidelines. CLNB should be safe if appropriate recommendations are made, which may enable patients to avoid overtreatment and unnecessary treatment‐related side effects.

An early study by Cai et al. included 702 NPC patients from China diagnosed between 1958 and 1972, 31% of them had CLNB.^12^ They found that partial excision of the movable lymph nodes has associated with an inferior survival compared to those receiving complete excision (22% vs. 50%).^12^ The study from Dickson included 209 patients in the United States between 1950 and 1976 (49% were Chinese), 34.0% of patients had CLNB, and patients receiving CLNB before primary radiotherapy have poorer survival than the non‐biopsied group (25% vs. 46.9%).^13^ However, chemotherapy in the above two studies is not the main component of the definitive treatment of NPC. With the advancement of imaging technology, it is believed that fewer patients have undergone CLNB. In addition, the use of chemotherapy has been confirmed to markedly decreased the incidence of distant metastasis and improve radiosensitivity.^29^ A recent study from Sun Yat‐sen University Cancer Center included 1492 NPC patients who were treated with IMRT, 12.3% of patients had CLNB, the results showed that CLNB was not associated with inferior survival outcomes before and after PSM.^10^ Another study included 2910 NPC patients from the SEER program, including 416 (14.3%) patients who underwent CLNB, and they found that CLNB was not related to impaired survival outcomes in the entire cohort, while an inferior prognosis of CLNB was found for patients with WHO II subtype.^11^ However, 1321 (45.4%) patients had metastasis disease and the chemotherapy use was not included in the above study,^11^ which limited the representativeness of this study to the general population. In our study, 16.9% of patients had CLNB, and our study also showed that patients receiving CLNB were not associated with inferior survival outcomes. Therefore, CLNB will not adversely affect the prognosis in the era of multimodal therapy of NPC.

No significant difference in survival between those with and without CLNB may be due to a higher percentage of chemotherapy use in patients who received CLNB. In the study by Yang et al., 14.8% of patients who had CLNB receiving induction chemotherapy, which was significantly higher than those without CLNB (9.2%) (*p* = 0.001). However, the percentage of concurrent chemotherapy was not significantly different.^10^ In our study, most of the patients (94.8%) receiving chemotherapy, while the addition of CLNB was not associated with a higher percentage of chemotherapy use (*p* = 0.950). The widespread use of chemotherapy, MRI, and IMRT has been confirmed to significantly decrease the incidence of distant metastasis and improved survival outcomes.^14^, ^15^, ^16^, ^29^ As CLNB would not contribute to inferior survival outcomes, more aggressive therapy may not be required.

In our previous study, we have found that histology was the independent prognostic factor for NPC.^30^ A previous SEER study showed that CLNB was associated with an inferior survival for the WHO II subtype.^11^ As we mentioned before, 45.4% of patients in their study had metastasis disease and 13.6% of patients did not receive radiotherapy.^11^ Therefore, it is difficult to accurately assess the impact of CLNB on patient survival. In our study, no association was found between CLNB and patient survival after stratification by histology.

Nasopharyngeal carcinoma is a disease with a high incidence among the Chinese population.^2^, ^3^ Despite immigrating to the United States, the Chinese population in the United States is still a high‐risk group of NPC.^31^, ^32^ In our study, we found that the Chinese population had a lower percentage of CLNB compared to their White Americans counterpart. This may be due to differences in morbidity leading to different diagnostic preferences by oncologists. NPC patients have a high probability of CLNM.^4^ Therefore, for those with CLNM, oncologists should also pay attention to the assessment of the nasopharynx during diagnostic evaluation in different races/ethnicities. Regarding the survival outcome, we also found that the receipt of CLNB was not associated with inferior survival after stratification by histology.

Several limitations should be acknowledged in the current study. First, the retrospective nature of our study may be limited by selection bias. Second, the SEER program does not provide information regarding the regimen and sequence of chemotherapy, the intensity of chemotherapy, the technique, and dose of radiotherapy, EBV status, and comorbidities. Third, SEER lacks data regarding locoregional recurrence or distant metastasis after treatment, both of which represent an important indicator in the assessment of prognosis and areas for future research on this topic. Moreover, the SEER database included patients from a long time period, during which the treatment mode of NPC had changed a lot. However, the stratified analyses by the year of diagnosis were conducted and similar results were found in our study. Finally, NPC is less prevalent in the United States and WHO type I NPC is the predominant histologic subtype in the US (in our study, 40.2%, 32.4%, and 27.4% of patients were WHO I, WHO II, and WHO III subtypes, respectively), so the findings in the present study may not be generalizable to the clinical practice in whole populations, especially for those living in a highly endemic area such as Southern China. However, two studies including NPC patients from Southern China also showed that pretreatment CLNB was not a significant determinant for distant metastases and mortality (4.8% with WHO I‐II subtype and 95.2% with WHO III subtype).^10^, ^33^ The primary strength of our study is that we used a large population‐based cohort to better reflect the real‐world clinical practice because a randomized controlled trial is impossible to address the effect of CLNB on prognosis for this patient subset.

In conclusion, additional CLNB is not associated with an inferior survival outcome in NPC. Our study provides a reference for the clinical practice of NPC.

## CONFLICT OF INTEREST

The authors declare no conflict of interest.

## ETHICS STATEMENT

Because the SEER program is a de‐identified database, the institutional review board of the First Affiliated Hospital of Xiamen University determined the current study to be exempt from review.

## Data Availability

The dataset analyzed during the current study is available in the Surveillance, Epidemiology, and End Results (SEER) database and can be accessed in detail through the utilization of SEER*Stat (https://seer.cancer.gov/data/).
